# The extensive and functionally uncharacterized mitochondrial phosphoproteome

**DOI:** 10.1016/j.jbc.2021.100880

**Published:** 2021-06-16

**Authors:** Natalie M. Niemi, David J. Pagliarini

**Affiliations:** 1 Department of Biochemistry & Molecular Biophysics, Washington University in St Louis, St Louis, Missouri, USA; 2 Departments of Cell Biology and Physiology, Biochemistry & Molecular Biophysics, and Genetics, Washington University in St Louis, St Louis, Missouri, USA; 3 Morgridge Institute for Research, Madison, Wisconsin, USA; 4 Department of Biochemistry, University of Madison-Wisconsin, Madison, Wisconsin, USA

**Keywords:** mitochondria, protein phosphorylation, protein phosphatase, protein kinase, phosphoproteomics, AKAP, A-kinase anchoring protein, BCKDK, branched chain amino acid dehydrogenase kinase, PDH, pyruvate dehydrogenase, PDK, pyruvate dehydrogenase kinase, PKL, protein kinase-like, PTM, posttranslational modification, MS, mass spectrometry, MTS, mitochondrial targeting sequence

## Abstract

More than half a century ago, reversible protein phosphorylation was linked to mitochondrial metabolism through the regulation of pyruvate dehydrogenase. Since this discovery, the number of identified mitochondrial protein phosphorylation sites has increased by orders of magnitude, driven largely by technological advances in mass spectrometry-based phosphoproteomics. However, the majority of these modifications remain uncharacterized, rendering their function and relevance unclear. Nonetheless, recent studies have shown that disruption of resident mitochondrial protein phosphatases causes substantial metabolic dysfunction across organisms, suggesting that proper management of mitochondrial phosphorylation is vital for organellar and organismal homeostasis. While these data suggest that phosphorylation within mitochondria is of critical importance, significant gaps remain in our knowledge of how these modifications influence organellar function. Here, we curate publicly available datasets to map the extent of protein phosphorylation within mammalian mitochondria and to highlight the known functions of mitochondrial-resident phosphatases. We further propose models by which phosphorylation may affect mitochondrial enzyme activities, protein import and processing, and overall organellar homeostasis.

## Phosphorylation and mitochondrial metabolism

Metabolism must respond to dynamic shifts in nutrient availability and energy demands. Phosphorylation, being a rapid and reversible posttranslational modification (PTM), is well suited to calibrate these needs. Indeed, phosphorylation has long been known to modulate metabolic signaling: the first protein associated with phosphorylation-based regulation was glycogen phosphorylase (1)—a discovery first reported in 1955 by Edwin Krebs and Edmond Fischer that earned them the 1992 Nobel Prize in Physiology and Medicine (2).

Early observations also linked phosphorylation to mitochondria. Albert Lehninger's group noted in the mid-1940s that incorporation of radioactive phosphate into rat liver phosphoproteins was dependent upon oxidative phosphorylation (3), and Eugene Kennedy and colleagues first demonstrated cellular kinase activity against casein using soluble mitochondrial extract in 1954 (4). However, the discovery of pyruvate dehydrogenase (PDH) regulation by phosphorylation in 1969 by Lester Reed's group (and Otto Wieland shortly thereafter) firmly established the role of this PTM in mitochondrial biology (5, 6).

**Figure 1:**
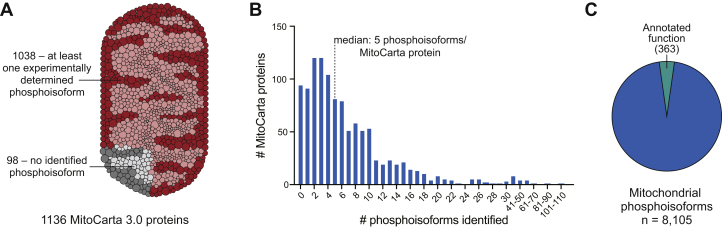
**Phosphorylation is prevalent on mitochondrial proteins.***A*, of the 1136 proteins identified in MitoCarta 3.0, 1038 have at least one experimentally determined phosphoisoform, while 98 have no reported phosphoisoforms per PhosphoSitePlus as of March 2021 (7). *B*, histogram analysis showing the number of phosphoisoforms identified per mitochondrial proteins; the median number of phosphoisoforms is shown with a dotted line. C. Graphical representation of all experimental identifications of mitochondrial phosphorylation events (8105) *versus* those with an annotated function (per the PhosphoSite Plus website). Notably, there is only ~4.5% of overlap.

Since these seminal discoveries, major technological advances in mass spectrometry (MS)-based quantitative phosphoproteomics have uncovered tens of thousands of phosphorylation sites on proteins across various cell types, tissues, and organisms (7). By curating many of these datasets, we found, perhaps surprisingly, that ∼91% of annotated mitochondrial proteins have at least one reported phosphorylation site as of March 2021 (Fig. 1*A*). Furthermore, these mitochondrial proteins have an average of approximately eight distinct phosphorylation sites (median = 5, Fig. 1*B*), demonstrating that this modification is highly prevalent across the mitochondrial proteome.

Are these modifications broadly meaningful for mitochondrial biology? The answer has remained unclear for some time. Selecting and biochemically characterizing individual phosphorylation events from these large, complex datasets has proven challenging, obscuring insight into how these modifications might directly alter mitochondrial protein function. Thus, despite their prevalence, less than 5% of these phosphorylated residues are associated with any published investigation (Fig. 1*C*), and even these few “characterized” modifications often lack critical details regarding function, stoichiometry (*i.e.*, the fraction of phosphorylated relative to unphosphorylated protein), and regulation across physiological contexts.

By some measures, it is reasonable to assume that these PTMs are a trivial distraction—biological “noise” captured by instruments with ever-increasing sensitivity. Indeed, systems-wide analyses have found that mitochondrial phosphoproteins typically exhibit low stoichiometry (8). Low-stoichiometry modifications need not be nonfunctional on principle; modifications that activate enzyme function, for example, can substantially alter biological function at low stoichiometry (9). Even for inhibitory modifications, the collective accumulation of low-stoichiometry events could decrease organellar function, as has been shown for mitochondrial protein acylation modifications (10, 11). Nonetheless, it is reasonable to assume that low-stoichiometry modifications are less likely to have biological impact and may reflect spurious events.

However, other observations suggest that these PTMs may possess important regulatory value. For example, multiple studies have found that phosphorylation sites on mitochondrial proteins are reproducibly dynamic across physiological states and models of disease (12, 13, 14, 15), implying functionality (or, at least, nonrandomness). Moreover, the select examples of purified mitochondrial phosphoproteins or phosphomimetics show that these PTMs certainly *can* alter protein activity when present (albeit, often at stoichiometry higher than what was observed in cells or tissues). Collectively, these observations demand better answers to two fundamental questions: are phosphorylation sites on mitochondrial proteins functionally meaningful? If so, which?

## Mitochondria-resident phosphatases

**Figure 2:**
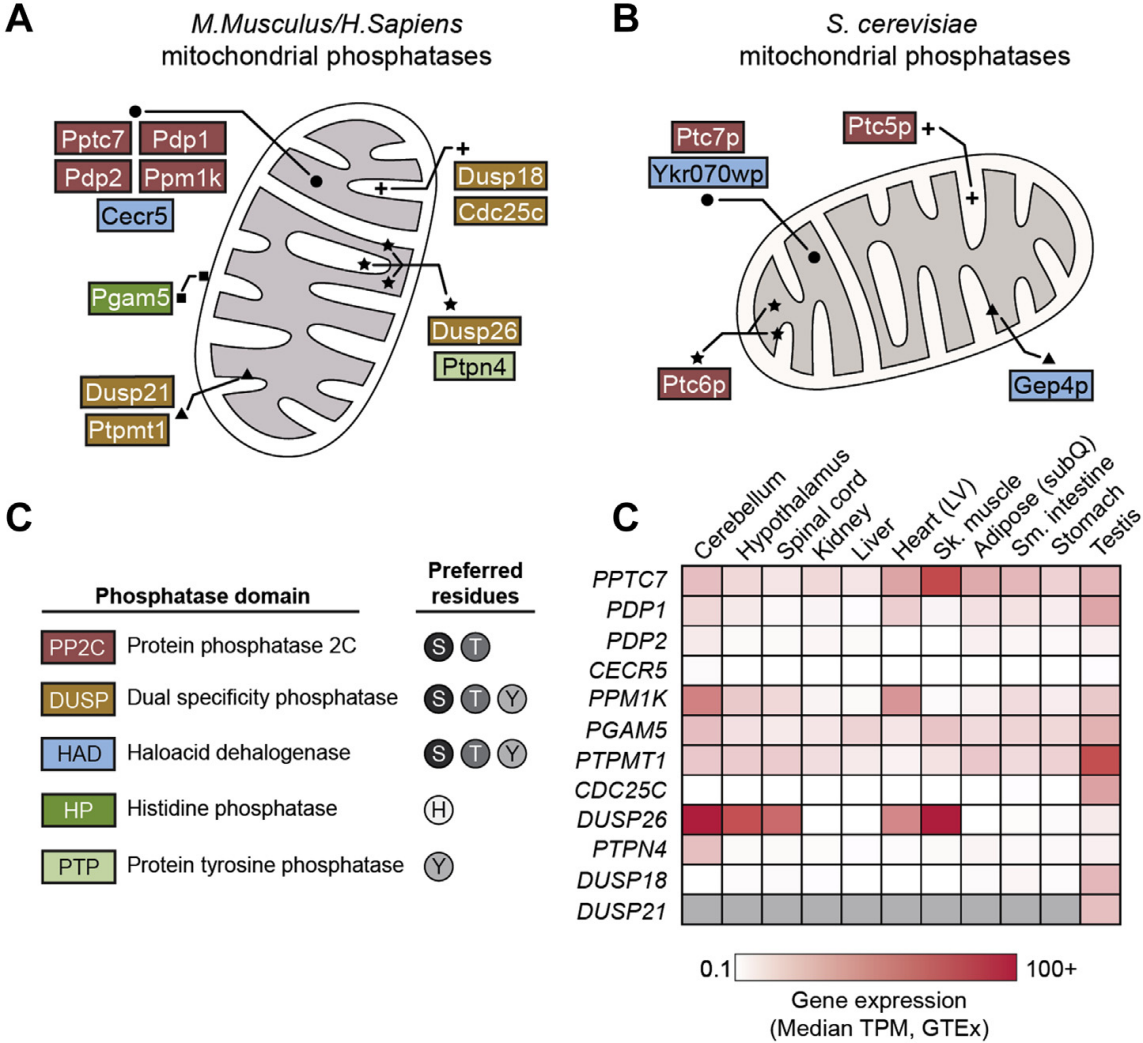
**Mitochondrial phosphatases identified across organisms.***A* and *B*, twelve phosphatases localize to mitochondria in mice and humans (*A*) and five reside in mitochondria in *Sacchraomyces cerevisiae* (*B*). Phosphatases are color-coded according to their phosphatase domain as defined in *panel C*. Sublocalization of mitochondrial phosphatases is denoted as follows: matrix localized = *circle*, inner mitochondrial membrane proteins = *triangle*, intermembrane space proteins = *cross*, outer membrane proteins = *square*, and dual-localized proteins, or proteins with unknown localization = *star*. *C*, proteins contain PP2C (*red*), DUSP (*orange*), HAD (*blue*) HP (*dark green*), and PTP (*light green*) phosphatase domains in mammalian mitochondria. *D*, gene expression data of mitochondrial phosphatases, represented as a heat map of median transcripts per million (TPM) as reported in the GTEx database (23). The heat map includes all ranges within 100 TPM, except for DUSP26, which has median TPM values of 104 for cerebellum and 193 for skeletal muscle. *Gray boxes* indicate transcripts were not quantified.

In debating the answers to the questions above, we were struck by another key data point: mitochondria appear to possess a set of resident protein phosphatases. The MitoCarta compendia have included 12 candidate protein phosphatases (16, 17, 18) (Fig. 2*A*), many of which are conserved across eukaryotic species, suggesting ancient origins for mitochondrial dephosphorylation (19) (Fig. 2*B*). The presence of 12 mitochondrial phosphatases in a single organelle may, at first glance, imply substantial functional redundancy, but evidence suggests that these proteins have distinct, nonoverlapping roles. First, mitochondrial phosphatases exploit five distinct catalytic domains, each with specific residue preferences that confer substrate selectivity (Fig. 2*C*). Furthermore, these phosphatases are subcompartmentalized across the organelle (20, 21, 22), physically dividing substrate pools and functions. RNA-seq profiles of many mitochondrial phosphatases show tissue-specific expression (23), further diversifying functions *in vivo* (Fig. 2*D*). Finally, genetic or pharmacological perturbation of many mitochondrial phosphatases results in distinct—and often severe—phenotypes, suggesting that these enzymes enable mitochondrial homeostasis through unique functions.

Given these insights, our approach to addressing the questions above has been to profile the mitochondrial phosphoproteome following the perturbation of one of these phosphatases (24, 25, 26). This approach can begin to resolve two fundamental challenges to understanding the mitochondrial phosphorylation network. First, these experiments identify phosphorylation sites that change in occupancy, suggesting functional relevance. This is a much needed prioritization metric for transitioning from high-throughput datasets to mechanistic investigations. Second, these studies place mitochondrial phosphatases within a biological framework, illuminating both the function of the phosphatase itself and the pathways dynamically influenced by phosphorylation. Beyond these biological insights, the study of phosphatase knockout systems offers a technical strength in providing a gain-of-signal readout (*i.e.*, elevated phosphorylation levels), which can be detected and quantified more accurately than loss-of-signal approaches. Finally, recent compendia of mitochondrial proteins include more candidate protein phosphatases than protein kinases, suggesting that perturbation of these genes provides a more straightforward strategy for illuminating the mitochondrial phosphoproteome. This strategy has recently highlighted surprising and unexpected insights into the influence of mitochondrial phosphorylation on organellar function and metabolic homeostasis.

## Knockout models of mitochondrial phosphatases highlight critical organellar functions

Genetic knockout models have been characterized for four mitochondrial phosphatases in yeast (24, 25, 27), and four in mice—Pdp1, Ppm1k, Pgam5, and Pptc7. Knockout models of phosphatases classically associated with metabolism, such as Pdp1 and Ppm1k, are viable and have phenotypes that likely derive from their annotated functions, including altered blood glucose levels for *Pdp1*^−/−^ mice and elevated circulating branched chain amino acids for *Ppm1k*^−/−^ mice (28, 29). Knockout of *Pgam5* leads to multiple phenotypes, including a Parkinson's-like movement disorder and T cell dysfunction (30, 31). Notably, global phosphoproteomic analysis has not been performed on these models, suggesting that the full breadth of substrates and pathways affected by loss of these phosphatases is not yet known.

We recently generated a global knockout mouse of the matrix-localized phosphatase *Pptc7* (26). Strikingly, the loss of Pptc7 manifests in severe metabolic abnormalities, including hypoketotic hypoglycemia, leading to fully penetrant perinatal lethality within one day of birth. On a cellular level, loss of the phosphatase markedly diminished mitochondrial content, suggesting that dephosphorylation of mitochondrial proteins is somehow critical for maintaining a healthy mitochondrial population. While the biochemical details of how this occurs are not fully worked out, these data demonstrate that proper management of mitochondrial phosphorylation is essential for mammalian development and, we propose, is of far greater importance to the biology of this organelle than is currently appreciated.

The considerable phenotypes exhibited by the four mitochondrial phosphatase knockout models demonstrate that at least a portion of observed mitochondrial phosphorylation sites hold functional relevance and cannot be attributed to mere technical artifacts. These observations also add important context to the issue of low phosphosite stoichiometry noted above. First, the reason that mitochondrial phosphosites exhibit low stoichiometry may be that mitochondrial phosphatases constitutively keep them that way. In our study, the loss of a mitochondrial phosphatase caused elevated stoichiometry at select phosphorylation sites, which subsequently led to impaired organellar function. This is consistent with the handful of mitochondrial phosphosites studied *in vitro*, most of which were inhibitory to protein function. Second, these data suggest that performing phosphoproteomic profiling under physiological conditions whereby the expression of a select phosphatase is low (*e.g.*, altered nutrient conditions or elevated stress) could yield very different (*i.e.*, elevated) protein stoichiometry.

Moving forward, it is essential that we continue to progress from large-scale profiling to mechanistic investigations of individual phosphorylation events. It is important to note that in mitochondrial phosphatase knockout models, we currently do not understand whether the observed phenotypes result from one/a select handful of phosphorylation events, or if the broader accumulation of phosphorylation on mitochondrial proteins is somehow collectively detrimental. Relatedly, recent work has shown that matrix-localized phosphorylation promotes selective mitophagy or turnover of mitochondrial proteins (32), suggesting that the primary effects of phosphorylation might not lie at the level of individual protein activity. Further investigations into the mechanisms by which dysregulated phosphorylation disrupts mitochondrial function should be an area of active focus in the future.

## Where are the kinases?

The severe phenotypes of mismanaged protein phosphorylation in knockout models of protein phosphatases naturally lead to a second question: How do mitochondrial proteins become phosphorylated? Multiple studies have identified kinases such as PKA that translocate to mitochondria and have even proposed a complete mitochondria-specific signaling system comprising G-protein-coupled receptors and cAMP production (33, 34, 35, 36). However, the conditions under which PKA translocates into mitochondria are not fully worked out, with some studies suggesting that PKA is not present in the matrix of select cell types, but instead is only on the outer mitochondrial membrane bound to an A-kinase anchoring protein (AKAP) (36). Furthermore, if G-protein-coupled receptor-mediated PKA activation does occur in the mitochondrial matrix, the signals that selectively activate these intraorganellar pathways, as well as those that resolve such signals, are not fully understood. Beyond PKA, other predominantly nonmitochondrial kinases, such as Src family members, Aurora kinase, and EGFR, have been partially localized to inner mitochondrial compartments (37, 38, 39). However, similar to PKA, the mechanisms by which they translocate, the signals prompting this translocation, and the systems underlying their organellar-specific regulation are not known.

Alternatively, it is possible that protein kinases long known to be resident in the mitochondrial matrix, such as the PDH kinases (PDKs) and the branched chain amino acid dehydrogenase (BCKDH) kinase (BCKDK), have broader substrate profiles than currently appreciated. These kinases are classically thought to have limited protein kinase activity across the mitochondrion due to their physical association with their respective dehydrogenase complexes (5, 40, 41). To our knowledge, this assumption has not been thoroughly tested, and some observations suggest that the PDKs and BCKDK may have a more diverse substrate pool than PDH and BCKDH. First, proximity labeling studies have shown at least three PDK isoforms with non-PDH interactors (42), challenging the notion that the PDKs are sequestered to the PDH complex. Second, treatment of porcine heart mitochondria with the pan-PDK inhibitor dichloroacetate and pyruvate diminished ^32^P-incorporation into multiple unidentified proteins, consistent with a model in which PDK isoforms phosphorylate proteins beyond PDH (43). Finally, recent work identified an unexpected role for BCKDK in the phosphorylation and regulation of ACLY—a cytosolic enzyme involved in fatty acid synthesis (44). Thus, noncanonical roles for the PDKs and BCKDK—both within and outside of mitochondria—seem likely and may influence mitochondrial phosphorylation profiles more than currently appreciated.

A third possibility invokes the action of resident atypical or metabolic kinases in phosphorylating mitochondrial proteins. Mammalian mitochondria contain five atypical “ADCK” kinases, which are members of the protein kinase-like (PKL) superfamily (45). Early on, one of these proteins (Coq8 in *Saccharomyces cerevisiae*) was linked to coenzyme Q biosynthesis and was postulated to directly phosphorylate proteins within the coenzyme Q pathway (46). However, structural analysis revealed that ADCK proteins possess multiple features poised to inhibit protein kinase activity, including an N-terminal domain that occupies the typical substrate-binding pocket (45, 47). Recent studies suggest that ADCKs more likely function as ATPases (48), although Coq8 and its mammalian orthologs are the only ADCKs that have been tested rigorously *in vitro* and *in vivo*. Beyond the ADCKs, 24 other small-molecule kinases reside within mitochondria that phosphorylate nucleotides (*e.g.*, the adenylate kinases AK2-4), lipids (*e.g.*, sphingosine kinase, SPHK2), metabolites (*e.g.*, phosphoenolcarboxykinase 2, PCK2), or unknown substrate classes (*e.g.*, ACAD10 and ACAD11). Emerging evidence suggests that metabolic kinases may moonlight as protein kinases (49), and thus careful biochemical studies are needed to determine whether these kinases may contribute to mitochondrial protein phosphorylation.

**Figure 3:**
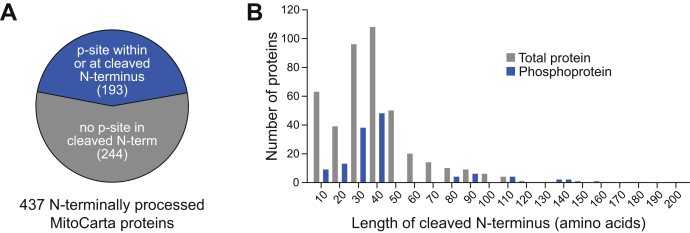
**Numerous mitochondrial proteins are phosphorylated near their N-termini.***A*, N-terminal cleavage sites that likely reflect a mitochondrial targeting sequence (MTS) were determined for 437 proteins. Of these, 193, or 44.2%, are phosphorylated within or proximal to their N-terminal cleaved region. *B*, distribution of lengths of cleaved N-termini of 437 proteins, binned by ten amino acid groups (*e.g.*, 1–10 amino acids). *Gray bars* reflect distribution of all 437 proteins by cleaved N-terminal length, *blue bars* reflect distribution of proteins phosphorylated within their cleaved N-terminus.

A final model posits that resident mitochondrial protein kinases are not needed at all. In our Pptc7 work, we identified elevated occupancy on phosphorylation sites at or within the mitochondrial targeting sequence (MTS) of multiple precursor proteins (26). The MTS—a short, N-terminal segment found on many mitochondrial precursors—is removed upon entry to the organelle and is thus unlikely to influence mature protein function. Despite this, more than 40% of MTS-containing mitochondrial proteins are phosphorylated within or proximal to this sequence (Fig. 3). These data suggest that phosphorylation may occur by cytosolic kinases prior to protein import and thus may influence the mitochondrial import and/or processing of precursor proteins. If phosphorylated mitochondrial precursors can be imported into mitochondria (as, it seems, they can in plastids (50)), this phenomenon might extend to much of the observed mitochondrial phosphoproteome. This mechanism would allow cross-compartmental regulation of mitochondrial phosphorylation, as cytosolic kinases, activated in response to various cellular cues, could phosphorylate mitochondrial precursors to affect their organellar targeting and function. This could provide a rapid and reversible mechanism to enrich specific molecular targets within mitochondria under dynamic cellular conditions. Furthermore, this mechanism could explain two current mysteries in the field of mitochondrial phosphorylation: (1) the paucity of mitochondrial-localized kinases relative to mitochondrial phosphatases, and (2) at least for MTS phosphorylation, the low stoichiometry, because they are inherently transient and resolved after import.

## Future directions and conclusions

The past decade has brought forth an incredible surge in the quality and quantity of phosphoproteomic studies. These studies, which span cellular and animal models, have revealed the unexpected prevalence of phosphorylation on mitochondrial proteins. However, questions remain regarding which of these modifications influence organellar function and the physiological conditions under which proteins become phosphorylated within the organelle.

Important next steps in understanding the systems-wide effects of phosphorylation in mitochondria include profiling the functions of mitochondrial phosphatases to understand not only their substrates and molecular functions but also how these translate into physiological dysfunction across organisms. Of the 12 known mitochondrial phosphatases, six (*i.e.*, Cecr5, Dusp18, Dusp21, Dusp26, Cdc25c, and Ptpn4) have no known substrates within mitochondria. We propose that a concerted effort to profile phosphatase knockout models with quantitative phosphoproteomic and metabolomic techniques will be key to establishing clear phosphatase–substrate relationships and for prioritizing biologically relevant modifications that affect mitochondrial processes. Furthermore, understanding the contexts under which mitochondrial phosphatases are regulated will elucidate a broader mitochondrial signaling network and help clarify how dynamic phosphorylation coordinates organellar function.

Finally, understanding mitochondrial phosphorylation may lead to new therapeutic options for elevating mitochondrial metabolism. Recent studies have already shown that kinase inhibition can alleviate mitochondrial dysfunction in mouse models of mitochondrial disease by dampening mTOR, PKC, or AMPK signaling (51, 52, 53). While it remains unclear how and to what extent the mitochondrial phosphoproteome is affected in each of these studies, these data collectively suggest that protein phosphorylation critically regulates mitochondrial function and may have therapeutic implications if properly exploited.

Mounting data suggest that reversible phosphorylation may be a widespread and underappreciated means of regulation within mitochondria and that its mismanagement could be an important underlying feature of mitochondrial pathophysiology. Rigorous new efforts to understand which mitochondrial proteins are affected by phosphorylation, to establish mechanistically how it alters their function, and to identify the enzymes that manage these modifications could motivate a powerful new therapeutic strategy: the control of mitochondrial metabolism *via* manipulation of intraorganellar phosphorylation networks.

## Conflict of interest

The authors declare that they have no conflicts of interest with the contents of this article.

## References

[1] Fischer E.H., Krebs E.G. (1955). Conversion of phosphorylase b to phosphorylase a in muscle extracts. J. Biol. Chem..

[2] Cohen P. (2002). The origins of protein phosphorylation. Nat. Cell Biol..

[3] Friedkin M., Lehninger A.L. (1949). Oxidation-coupled incorporation of inorganic radiophosphate into phospholipide and nucleic acid in a cell-free system. J. Biol. Chem..

[4] Burnett G., Kennedy E.P. (1954). The enzymatic phosphorylation of proteins. J. Biol. Chem..

[5] Linn T.C., Pettit F.H., Reed L.J. (1969). Alpha-keto acid dehydrogenase complexes. X. Regulation of the activity of the pyruvate dehydrogenase complex from beef kidney mitochondria by phosphorylation and dephosphorylation. Proc. Natl. Acad. Sci. U. S. A..

[6] Wieland O., Siess E. (1970). Interconversion of phospho- and dephospho- forms of pig heart pyruvate dehydrogenase. Proc. Natl. Acad. Sci. U. S. A..

[7] Hornbeck P.V., Zhang B., Murray B., Kornhauser J.M., Latham V., Skrzypek E. (2015). PhosphoSitePlus, 2014: Mutations, PTMs and recalibrations. Nucleic Acids Res..

[8] Wu R., Haas W., Dephoure N., Huttlin E.L., Zhai B., Sowa M.E., Gygi S.P. (2011). A large-scale method to measure absolute protein phosphorylation stoichiometries. Nat. Methods.

[9] Prus G., Hoegl A., Weinert B.T., Choudhary C. (2019). Analysis and interpretation of protein post-translational modification site stoichiometry. Trends Biochem. Sci..

[10] Weinert B.T., Moustafa T., Iesmantavicius V., Zechner R., Choudhary C. (2015). Analysis of acetylation stoichiometry suggests that SIRT3 repairs nonenzymatic acetylation lesions. EMBO J..

[11] Baeza J., Smallegan M.J., Denu J.M. (2016). Mechanisms and dynamics of protein acetylation in mitochondria. Trends Biochem. Sci..

[12] Grimsrud P.A., Carson J.J., Hebert A.S., Hubler S.L., Niemi N.M., Bailey D.J., Jochem A., Stapleton D.S., Keller M.P., Westphall M.S., Yandell B.S., Attie A.D., Coon J.J., Pagliarini D.J. (2012). A quantitative map of the liver mitochondrial phosphoproteome reveals posttranslational control of ketogenesis. Cell Metab..

[13] Zhao X., Leon I.R., Bak S., Mogensen M., Wrzesinski K., Hojlund K., Jensen O.N. (2011). Phosphoproteome analysis of functional mitochondria isolated from resting human muscle reveals extensive phosphorylation of inner membrane protein complexes and enzymes. Mol. Cell Proteomics.

[14] Deng N., Zhang J., Zong C., Wang Y., Lu H., Yang P., Wang W., Young G.W., Wang Y., Korge P., Lotz C., Doran P., Liem D.A., Apweiler R., Weiss J.N. (2011). Phosphoproteome analysis reveals regulatory sites in major pathways of cardiac mitochondria. Mol. Cell Proteomics.

[15] Reinders J., Wagner K., Zahedi R.P., Stojanovski D., Eyrich B., van der Laan M., Rehling P., Sickmann A., Pfanner N., Meisinger C. (2007). Profiling phosphoproteins of yeast mitochondria reveals a role of phosphorylation in assembly of the ATP synthase. Mol. Cell Proteomics.

[16] Pagliarini D.J., Calvo S.E., Chang B., Sheth S.A., Vafai S.B., Ong S.E., Walford G.A., Sugiana C., Boneh A., Chen W.K., Hill D.E., Vidal M., Evans J.G., Thorburn D.R., Carr S.A. (2008). A mitochondrial protein compendium elucidates complex I disease biology. Cell.

[17] Calvo S.E., Clauser K.R., Mootha V.K. (2016). MitoCarta2.0: An updated inventory of mammalian mitochondrial proteins. Nucleic Acids Res..

[18] Rath S., Sharma R., Gupta R., Ast T., Chan C., Durham T.J., Goodman R.P., Grabarek Z., Haas M.E., Hung W.H.W., Joshi P.R., Jourdain A.A., Kim S.H., Kotrys A.V., Lam S.S. (2021). MitoCarta3.0: An updated mitochondrial proteome now with sub-organelle localization and pathway annotations. Nucleic Acids Res..

[19] Frankovsky J., Vozarikova V., Nosek J., Tomaska L. (2021). Mitochondrial protein phosphorylation in yeast revisited. Mitochondrion.

[20] Hung V., Zou P., Rhee H.W., Udeshi N.D., Cracan V., Svinkina T., Carr S.A., Mootha V.K., Ting A.Y. (2014). Proteomic mapping of the human mitochondrial intermembrane space in live cells via ratiometric APEX tagging. Mol. Cell.

[21] Hung V., Lam S.S., Udeshi N.D., Svinkina T., Guzman G., Mootha V.K., Carr S.A., Ting A.Y. (2017). Proteomic mapping of cytosol-facing outer mitochondrial and ER membranes in living human cells by proximity biotinylation. Elife.

[22] Rhee H.W., Zou P., Udeshi N.D., Martell J.D., Mootha V.K., Carr S.A., Ting A.Y. (2013). Proteomic mapping of mitochondria in living cells via spatially restricted enzymatic tagging. Science.

[23] Carithers L.J., Ardlie K., Barcus M., Branton P.A., Britton A., Buia S.A., Compton C.C., DeLuca D.S., Peter-Demchok J., Gelfand E.T., Guan P., Korzeniewski G.E., Lockhart N.C., Rabiner C.A., Rao A.K. (2015). A novel approach to high-quality postmortem tissue procurement: The GTEx project. Biopreserv Biobank.

[24] Guo X., Niemi N.M., Hutchins P.D., Condon S.G.F., Jochem A., Ulbrich A., Higbee A.J., Russell J.D., Senes A., Coon J.J., Pagliarini D.J. (2017). Ptc7p dephosphorylates select mitochondrial proteins to enhance metabolic function. Cell Rep..

[25] Guo X., Niemi N.M., Coon J.J., Pagliarini D.J. (2017). Integrative proteomics and biochemical analyses define Ptc6p as the Saccharomyces cerevisiae pyruvate dehydrogenase phosphatase. J. Biol. Chem..

[26] Niemi N.M., Wilson G.M., Overmyer K.A., Vogtle F.N., Myketin L., Lohman D.C., Schueler K.L., Attie A.D., Meisinger C., Coon J.J., Pagliarini D.J. (2019). Pptc7 is an essential phosphatase for promoting mammalian mitochondrial metabolism and biogenesis. Nat. Commun..

[27] Osman C., Haag M., Wieland F.T., Brugger B., Langer T. (2010). A mitochondrial phosphatase required for cardiolipin biosynthesis: The PGP phosphatase Gep4. EMBO J..

[28] Dickinson M.E., Flenniken A.M., Ji X., Teboul L., Wong M.D., White J.K., Meehan T.F., Weninger W.J., Westerberg H., Adissu H., Baker C.N., Bower L., Brown J.M., Caddle L.B., Chiani F. (2016). High-throughput discovery of novel developmental phenotypes. Nature.

[29] Lu G., Sun H., She P., Youn J.Y., Warburton S., Ping P., Vondriska T.M., Cai H., Lynch C.J., Wang Y. (2009). Protein phosphatase 2Cm is a critical regulator of branched-chain amino acid catabolism in mice and cultured cells. J. Clin. Invest..

[30] Lu W., Karuppagounder S.S., Springer D.A., Allen M.D., Zheng L., Chao B., Zhang Y., Dawson V.L., Dawson T.M., Lenardo M. (2014). Genetic deficiency of the mitochondrial protein PGAM5 causes a Parkinson's-like movement disorder. Nat. Commun..

[31] Panda S., Srivastava S., Li Z., Vaeth M., Fuhs S.R., Hunter T., Skolnik E.Y. (2016). Identification of PGAM5 as a mammalian protein histidine phosphatase that plays a central role to negatively regulate CD4(+) T cells. Mol. Cell.

[32] Kolitsida P., Zhou J., Rackiewicz M., Nolic V., Dengjel J., Abeliovich H. (2019). Phosphorylation of mitochondrial matrix proteins regulates their selective mitophagic degradation. Proc. Natl. Acad. Sci. U. S. A..

[33] Hebert-Chatelain E., Desprez T., Serrat R., Bellocchio L., Soria-Gomez E., Busquets-Garcia A., Pagano Zottola A.C., Delamarre A., Cannich A., Vincent P., Varilh M., Robin L.M., Terral G., Garcia-Fernandez M.D., Colavita M. (2016). A cannabinoid link between mitochondria and memory. Nature.

[34] Ould Amer Y., Hebert-Chatelain E. (2018). Mitochondrial cAMP-PKA signaling: What do we really know?. Biochim. Biophys. Acta Bioenerg..

[35] Acin-Perez R., Salazar E., Kamenetsky M., Buck J., Levin L.R., Manfredi G. (2009). Cyclic AMP produced inside mitochondria regulates oxidative phosphorylation. Cell Metab..

[36] Lefkimmiatis K., Leronni D., Hofer A.M. (2013). The inner and outer compartments of mitochondria are sites of distinct cAMP/PKA signaling dynamics. J. Cell Biol..

[37] Hebert-Chatelain E., Jose C., Gutierrez Cortes N., Dupuy J.W., Rocher C., Dachary-Prigent J., Letellier T. (2012). Preservation of NADH ubiquinone-oxidoreductase activity by Src kinase-mediated phosphorylation of NDUFB10. Biochim. Biophys. Acta.

[38] Bertolin G., Bulteau A.L., Alves-Guerra M.C., Burel A., Lavault M.T., Gavard O., Le Bras S., Gagne J.P., Poirier G.G., Le Borgne R., Prigent C., Tramier M. (2018). Aurora kinase A localises to mitochondria to control organelle dynamics and energy production. Elife.

[39] Boerner J.L., Demory M.L., Silva C., Parsons S.J. (2004). Phosphorylation of Y845 on the epidermal growth factor receptor mediates binding to the mitochondrial protein cytochrome c oxidase subunit II. Mol. Cell Biol..

[40] Phillips D., Aponte A.M., Covian R., Balaban R.S. (2011). Intrinsic protein kinase activity in mitochondrial oxidative phosphorylation complexes. Biochemistry.

[41] Harris R.A., Bowker-Kinley M.M., Huang B., Wu P. (2002). Regulation of the activity of the pyruvate dehydrogenase complex. Adv. Enzyme Regul..

[42] Antonicka H., Lin Z.Y., Janer A., Aaltonen M.J., Weraarpachai W., Gingras A.C., Shoubridge E.A. (2020). A high-density human mitochondrial proximity interaction network. Cell Metab..

[43] Aponte A.M., Phillips D., Harris R.A., Blinova K., French S., Johnson D.T., Balaban R.S. (2009). 32P labeling of protein phosphorylation and metabolite association in the mitochondria matrix. Methods Enzymol..

[44] White P.J., McGarrah R.W., Grimsrud P.A., Tso S.C., Yang W.H., Haldeman J.M., Grenier-Larouche T., An J., Lapworth A.L., Astapova I., Hannou S.A., George T., Arlotto M., Olson L.B., Lai M. (2018). The BCKDH kinase and phosphatase integrate BCAA and lipid metabolism via regulation of ATP-citrate lyase. Cell Metab..

[45] Stefely J.A., Reidenbach A.G., Ulbrich A., Oruganty K., Floyd B.J., Jochem A., Saunders J.M., Johnson I.E., Minogue C.E., Wrobel R.L., Barber G.E., Lee D., Li S., Kannan N., Coon J.J. (2015). Mitochondrial ADCK3 employs an atypical protein kinase-like fold to enable coenzyme Q biosynthesis. Mol. Cell.

[46] Xie L.X., Hsieh E.J., Watanabe S., Allan C.M., Chen J.Y., Tran U.C., Clarke C.F. (2011). Expression of the human atypical kinase ADCK3 rescues coenzyme Q biosynthesis and phosphorylation of Coq polypeptides in yeast coq8 mutants. Biochim. Biophys. Acta.

[47] Stefely J.A., Licitra F., Laredj L., Reidenbach A.G., Kemmerer Z.A., Grangeray A., Jaeg-Ehret T., Minogue C.E., Ulbrich A., Hutchins P.D., Wilkerson E.M., Ruan Z., Aydin D., Hebert A.S., Guo X. (2016). Cerebellar ataxia and coenzyme Q deficiency through loss of unorthodox kinase activity. Mol. Cell.

[48] Reidenbach A.G., Kemmerer Z.A., Aydin D., Jochem A., McDevitt M.T., Hutchins P.D., Stark J.L., Stefely J.A., Reddy T., Hebert A.S., Wilkerson E.M., Johnson I.E., Bingman C.A., Markley J.L., Coon J.J. (2018). Conserved lipid and small-molecule modulation of COQ8 reveals regulation of the ancient kinase-like UbiB family. Cell Chem Biol..

[49] Lu Z., Hunter T. (2018). Metabolic kinases moonlighting as protein kinases. Trends Biochem. Sci..

[50] Waegemann K., Soll J. (1996). Phosphorylation of the transit sequence of chloroplast precursor proteins. J. Biol. Chem..

[51] Johnson S.C., Yanos M.E., Kayser E.B., Quintana A., Sangesland M., Castanza A., Uhde L., Hui J., Wall V.Z., Gagnidze A., Oh K., Wasko B.M., Ramos F.J., Palmiter R.D., Rabinovitch P.S. (2013). mTOR inhibition alleviates mitochondrial disease in a mouse model of Leigh syndrome. Science.

[52] Martin-Perez M., Grillo A.S., Ito T.K., Valente A.S., Han J., Entwisle S.W., Huang H.Z., Kim D., Yajima M., Kaeberlein M., Villen J. (2020). PKC downregulation upon rapamycin treatment attenuates mitochondrial disease. Nat. Metab..

[53] Zaninello M., Palikaras K., Naon D., Iwata K., Herkenne S., Quintana-Cabrera R., Semenzato M., Grespi F., Ross-Cisneros F.N., Carelli V., Sadun A.A., Tavernarakis N., Scorrano L. (2020). Inhibition of autophagy curtails visual loss in a model of autosomal dominant optic atrophy. Nat. Commun..

